# Periorbital Fat Loss From Prostaglandin Analogues: Dermatologic and Cosmetic Considerations

**DOI:** 10.1111/jocd.71254

**Published:** 2026-10-30

**Authors:** Lily Kaufman, Aliza Khuhro, Sierra Schwierking, Lilia Valentic, Sonja Kobayashi, Lauren Malley, Olivia Ueltschi, Lucy Rose, Brittany Dulmage

**Affiliations:** ^1^ The Ohio State University College of Medicine Columbus Ohio USA; ^2^ Department of Dermatology The Ohio State University Wexner Medical Center Columbus Ohio USA

**Keywords:** periorbital fat loss, prostaglandin analogue, prostaglandin‐associated periorbitopathy

## Abstract

**Background:**

Topical prostaglandin (PG) analogues, originally developed as first‐line therapies for glaucoma, are increasingly used in dermatology and cosmetic settings to promote eyelash and eyebrow growth. However, their use has been associated with prostaglandin‐associated periorbitopathy (PAP), a constellation of adverse effects including periorbital fat atrophy, deepening of the upper eyelid sulcus, ptosis, and skin hyperpigmentation. These changes can be subtle but carry significant aesthetic and psychosocial consequences, particularly in patients using PG analogues for cosmetic enhancement.

**Aims:**

This review aims to synthesize current knowledge on the clinical features, pathophysiology, and reversibility of PAP, with an emphasis on implications for dermatologic practice.

**Methods:**

A narrative review of the literature was conducted using PubMed, focusing on dermatologic, ophthalmologic, and cosmetic reports related to PG analogue use and PAP.

**Results:**

Although PAP is well recognized in ophthalmology, dermatology‐specific data remain sparse. Many over‐the‐counter eyelash serums contain PG analogues yet lack accurate labeling and regulatory oversight. Diagnostic delays are common, and dermatologists may overlook serum use as a contributing factor in periorbital changes.

**Conclusions:**

As the off‐label and consumer‐driven use of PG analogues continues to rise, dermatologists must remain vigilant in identifying PAP. Routine history‐taking should include inquiry about eyelash and eyebrow serums. Greater regulatory oversight, patient education, and dermatology‐focused research are needed to promote safe and informed use.

## 1 Introduction

Since their introduction in ophthalmology, topical prostaglandin (PG) analogues including latanoprost, travoprost, tafluprost, bimatoprost, and unoprostone have become first‐line therapies for glaucoma, valued for their efficacy and tolerability. In addition to lowering intraocular pressure, PG analogues may cause side effects such as eyelash trichomegaly and hypertrichosis [1], drawing interest for cosmetic applications. Since the Food and Drug Administration (FDA) approval of bimatoprost ophthalmic solution for eyelash hypotrichosis, PG analogues have been increasingly used in cosmetic dermatology and as supportive care for alopecia [2, 3, 4]. Eyelash serums have grown popular in both prescription and over‐the‐counter (OTC) forms, with or without PG analogues [4].

Prostaglandin‐associated periorbitopathy (PAP) is a recognized side effect of topical PG analogues, characterized by periorbital fat atrophy, eyelid sulcus deepening, elevation of the eyelid crease, enophthalmos, ptosis, skin darkening, ectropion, and exaggerated dermatochalasis. These changes may negatively affect facial appearance and have been associated with psychosocial consequences [5, 6, 7, 8, 9, 10, 11, 12, 13, 14, 15, 16, 17, 18, 19, 20, 21, 22, 23, 24, 25, 26, 27]. Notably, the existing literature describing PAP has been generated primarily in the context of prescription ophthalmic PG analogues and prostamides [1, 6, 7, 9, 10, 11, 12, 13, 14, 15, 16, 17, 18, 19, 21, 22, 24, 25, 26]. In contrast, cosmetic eyelash serums often contain PG analogue derivatives rather than the approved ophthalmic agents, frequently with undisclosed concentrations and variable labeling. Although these compounds are pharmacologically related, they differ in formulation, regulation, and available safety data, which is important for accurate interpretation of reported risk [4]. With growing awareness beyond ophthalmology, this review synthesizes current evidence on aesthetic and functional impacts of PG analogues on the periorbital region, with relevance to dermatology practice and patient counseling.

## 2 Methods

This narrative review was informed by a literature search conducted in PubMed between June and July 2025. Search terms included combinations of the following keywords: *prostaglandin analogue, prostaglandin‐associated periorbitopathy, periorbital fat loss, periorbital fat atrophy, deepening of the upper eyelid sulcus, bimatoprost periorbitopathy, latanoprost periorbitopathy, prostaglandin eyelash serum, topical prostaglandin adipocyte*, and *prostaglandin orbital fat*.

English‐language publications were included without restriction on study design. All articles identified through the search strategy that addressed PAP were reviewed, and those deemed relevant to the clinical, mechanistic, or cosmetic aspects of PAP were included. Due to the limited size of the literature, this approach was intentionally inclusive to ensure comprehensive coverage. In total, approximately 57 articles were included, comprising a mix of basic science studies, animal models, clinical studies, and case reports or case series. Additional contextual information regarding cosmetic prostaglandin‐containing eyelash serums was obtained from publicly available manufacturer websites.

As this was a narrative review, no formal evidence‐grading system was applied. Where available, findings from larger clinical cohorts were emphasized; however, due to the relative scarcity of cosmetic‐focused data, conclusions were informed by the totality of available evidence.

## 3 Clinical Presentation

PAP presents with features including periorbital fat loss, deepening of the upper eyelid sulcus (DUES), enophthalmos, and ptosis. These aesthetic changes are most often seen in glaucoma patients. Bimatoprost 0.03% solution is currently the only FDA‐approved prostaglandin analogue for eyelash hypotrichosis, and it is thought to cause the most severe PAP of the prostaglandin analogues [28], with the highest incidence of PAP at 1 month compared to latanoprost and travoprost [9]. Some patients may find this side effect desirable, described as a bimatoprost‐induced “chemical blepharoplasty” [20]. Topical PG analogue use has been associated with DUES as early as 1 month after beginning therapy [29]. Dermatologists should be aware of the wide spectrum and severity of reported side effects, especially with the growing use of eyelash and eyebrow serums. A summary of clinical findings of PAP and associated agents is summarized in Table 1.

**TABLE 1:** Clinical findings of prostaglandin‐associated periorbitopathy, associated agents, and key references.

Clinical finding	Description	Associated drug(s)	Key studies/references
Periorbital Fat Loss	A reduction in the volume and thickness of the fat pads surrounding the eye orbit, especially the upper and lower eyelids	Bimatoprost [6, 9, 12] Travoprost [6, 9] Latanoprost [9]	[6]. Jayaprakasam A, Ghazi‐Nouri S. Periorbital fat atrophy ‐ an unfamiliar side effect of prostaglandin analogues. *Orbit*. 2010;29(6):357–359. doi:10.3109/01676830.2010.527028 [9]. Kucukevcilioglu M, Bayer A, Uysal Y, Altinsoy HI. Prostaglandin associated periorbitopathy in patients using bimatoprost, latanoprost and travoprost. *Clin Exp Ophthalmol*. 2014;42(2):126–131. doi:10.1111/ceo.12163 [12]. Filippopoulos T, Paula JS, Torun N, Hatton MP, Pasquale LR, Grosskreutz CL. Periorbital changes associated with topical bimatoprost. *Ophthalmic Plast Reconstr Surg*. 2008;24(4):302–307. doi:10.1097/IOP.0b013e31817d81df
Upper Eyelid Sulcus Deepening (DUES)	A deepening of the superior palpebral sulcus (upper eyelid crease) due to loss of orbital fat and tissue	Bimatoprost [29] Latanoprost [18, 22] (mild) Tafluprost [26]	[18]. Nakakura S, Yamamoto M, Terao E, et al. Prostaglandin‐associated periorbitopathy in latanoprost users. *Clin Ophthalmol*. 2014;9:51–56. Published 2014 Dec 30. doi:10.2147/OPTH.S75651 [22]. Sakata R, Shirato S, Miyata K, Aihara M. Incidence of deepening of the upper eyelid sulcus in prostaglandin‐associated periorbitopathy with a latanoprost ophthalmic solution. *Eye (Lond)*. 2014;28(12):1446–1451. doi:10.1038/eye.2014.224 [26]. Sakata R, Shirato S, Miyata K, Aihara M. Incidence of deepening of the upper eyelid sulcus on treatment with a tafluprost ophthalmic solution. *Jpn J Ophthalmol*. 2014;58(2):212–217. doi:10.1007/s10384‐013‐0299‐8 [29]. Aihara M, Shirato S, Sakata R. Incidence of deepening of the upper eyelid sulcus after switching from latanoprost to bimatoprost. *Jpn J Ophthalmol*. 2011;55(6):600–604. doi:10.1007/s10384‐011‐0075‐6
Enophthalmos	Posterior displacement of the eye into the orbit	Bimatoprost [6, 10, 21] Travoprost [6, 21] Latanoprost [21]	[6]. Jayaprakasam A, Ghazi‐Nouri S. Periorbital fat atrophy—an unfamiliar side effect of prostaglandin analogues. *Orbit*. 2010;29(6):357–359. doi:10.3109/01676830.2010.527028 [10]. Wang PX, Koh VT, Cheng JF. Periorbital muscle atrophy associated with topical bimatoprost therapy. *Clin Ophthalmol*. 2014;8:311–314. Published 2014 Jan 29. doi:10.2147/OPTH.S55754 [21]. Rabinowitz MP, Katz LJ, Moster MR, et al. Unilateral Prostaglandin‐Associated Periorbitopathy: A Syndrome Involving Upper Eyelid Retraction Distinguishable From the Aging Sunken Eyelid. *Ophthalmic Plast Reconstr Surg*. 2015;31(5):373–378. doi:10.1097/IOP.0000000000000351
Eyelid Retraction/Ptosis	Eyelid retraction: Abnormally elevated resting point of upper eyelid or depressed resting point of lower eyelid, exposing more of the sclera Ptosis: drooping of the upper eyelid	Bimatoprost [11, 30] Travoprost [11, 30] Latanoprost [11, 30]	[11]. Shah M, Lee G, Lefebvre DR, et al. A cross‐sectional survey of the association between bilateral topical prostaglandin analogue use and ocular adnexal features. *PLoS One*. 2013;8(5):e61638. Published 2013 May 1. doi:10.1371/journal.pone.0061638 [44]. Custer PL, Kent TL. Observations on Prostaglandin Orbitopathy. *Ophthalmic Plast Reconstr Surg*. 2016;32(2):102–105. doi:10.1097/IOP.0000000000000431
Pseudo‐Blepharoplasty Effect	Cosmetic appearance of eye mimicking surgical blepharoplasty (upper eyelid lift), due to fat atrophy and other structural changes of eyelid	Bimatoprost [14, 20]	[20]. Sarnoff DS, Gotkin RH. Bimatoprost‐induced chemical blepharoplasty. *J Drugs Dermatol*. 2015;14(5):472–477. [14]. Sira M, Verity DH, Malhotra R. Topical bimatoprost 0.03% and iatrogenic eyelid and orbital lipodystrophy. *Aesthet Surg J*. 2012;32(7):822–824. doi:10.1177/1090820X12455659
Cosmetic Asymmetry (Unilateral Use)	Facial asymmetry in eyes due to unilateral use of topical prostaglandins and subsequent periorbital changes	All PG analogues if used unilaterally [10, 25, 31]	[10]. Wang PX, Koh VT, Cheng JF. Periorbital muscle atrophy associated with topical bimatoprost therapy. *Clin Ophthalmol*. 2014;8:311–314. Published 2014 Jan 29. doi:10.2147/OPTH.S55754 [25]. Yip IYM, Ramasamy B. Unilateral orbital fat change. *BMJ Case Rep*. 2018;2018:bcr2017223525. Published 2018 Jan 4. doi:10.1136/bcr‐2017‐223 525 [50]. Abalo‐Lojo JM, Ferreiro PV, Asorey MK, Colmenero AE, Gonzalez F. Improvement of Prostaglandin‐Associated Periorbitopathy after Discontinuing Treatment. *Turk J Ophthalmol*. 2023;53(1):8–12. doi:10.4274/tjo.galenos.2022.24365

*Note:* The clinical features summarized in this table are supported predominantly by studies of ophthalmic PG analogue use in glaucoma populations. Evidence specific to cosmetic eyelash or eyebrow serum use is limited and largely confined to case reports.

Cosmetically concerning asymmetry from unilateral use affects nearly all ophthalmology patients with unilateral PG analogue treatment. While the primary goal of eyelash and eyebrow serums is to improve appearance, unexpected and unwanted PAP can be distressing. Few studies describe the extent of changes experienced by those specifically using prostaglandin analogues for eyelash or eyebrow regrowth, and most effects have been documented through case reports. One patient using Lash Boost (Rodan & Fields, San Francisco, CA) reported findings of thin, wrinkled, and darkened skin with periorbital hollowing following 10 months of use [5]. Another patient presented with greenish periorbital darkening after 1 year of PG analogue use [32]. These findings suggest that even an OTC “cosmetic” dose can produce significant adverse effects.

Although PAP is a clinical diagnosis, prior studies of PG analogue users have examined characteristic imaging findings. Magnetic resonance imaging (MRI) and computed tomography (CT) of patients with PAP have demonstrated orbital fat atrophy and enophthalmos, particularly apparent when asymmetry is present and the unaffected side serves as an internal control. MRI evidence of fat volume loss correlating with clinical asymmetry has been demonstrated in patients using unilateral prostaglandin analogues [6]. However, relevant findings on imaging may not be present in subtle cases; one CT‐based study failed to detect significant fat volume differences between treated and untreated eyes in glaucoma patients, highlighting the importance of careful clinical examination and history‐taking [13].

## 4 Mechanism of Action

PG analogues are structural derivatives of prostaglandin F2α (PGF2α) and act primarily through activation of the prostanoid FP receptor, a G‐protein‐coupled receptor involved in regulating smooth muscle tone, vascular permeability, and extracellular matrix remodeling [33]. FP receptors are also expressed in cutaneous tissues, including the hair follicle. In this setting, FP receptor activation prolongs the anagen (growth) phase of the hair cycle, contributing to the well‐documented side effects of trichomegaly and hypertrichosis observed during topical PG analogue use [34].

In addition to their effects on hair follicles, PGF2α analogues have been shown to influence adipocyte biology. FP receptor activation in adipose tissue inhibits adipocyte differentiation and promotes lipolysis, leading to a net reduction in fat volume [35]. In vitro studies have demonstrated that PGF2α can suppress the expression of key adipogenic transcription factors such as peroxisome proliferator‐activated receptor γ (PPARγ), impairing adipocyte maturation [36]. In vivo studies have also demonstrated a reduction in adipocyte count or fat pad volume following exposure to both topical [34] and locally injected PG analogues [37, 38, 39, 40], highlighting their potential to disrupt the homeostasis of orbital adipose tissue, even with topical or cosmetic use. These findings help explain the periorbital fat atrophy observed in PAP, suggesting that chronic exposure to topical PG analogues may disrupt the homeostasis of orbital adipose tissue. This underscores the importance of recognizing these effects when prescribing eyelash serums or evaluating patients with a history of PG analogue use.

In addition to their effects on adipose tissue, PG analogues may contribute to structural changes in the periorbital region through dysregulation of the extracellular matrix (ECM). The ECM is a network of proteins, including collagen and elastin, that provides structural support and regulates tissue remodeling throughout the body. In the periorbital region, ECM integrity helps maintain eyelid tone, skin elasticity, and fat pad positioning [41]. PG analogues may influence ECM remodeling by altering the balance between matrix metalloproteinases (MMPs), which degrade ECM components, and their tissue inhibitor metalloproteinases (TIMPs). Although some early hypotheses proposed that increased MMP activity could lead to collagen degradation and contribute to PAP, recent findings are mixed. A study analyzing periorbital muscle, adipose, and collagenous tissues in PG analogue users found decreased MMP‐3 expression in collagen, with this pattern correlating with clinical features such as fat atrophy and dermatochalasis. These results suggest that ECM dysregulation, rather than direct collagen degradation alone, may contribute to the complex tissue remodeling seen in PAP [41, 42].

## 5 Populations at Risk

Eyelash and eyebrow serums, available both by prescription and OTC, are increasingly used by healthy individuals and patients with alopecia to enhance the appearance of eyelashes and eyebrows. These products, often containing PG analogues, claim to promote eyelash strength, length, luster, and thickness, and are sometimes marketed for supporting regrowth after chemotherapy‐induced anagen effluvium or alopecia areata [4, 5, 43, 44]. Additionally, PG analogues are used off‐label in dermatology to augment treatment of various types of alopecia resulting in hypotrichosis, including scarring and non‐scarring alopecias [44]. As the use of PG‐containing products expands across both cosmetic and therapeutic settings, dermatologists should be aware of the potential for periorbital changes and ensure appropriate counseling for patients at risk of these side effects. A summary of commercially available PG analogue‐containing eyelash and eyebrow serums is summarized in Table 2.

**TABLE 2:** Overview of commercial eyelash and eyebrow serums containing prostaglandin analogues: Compound details and safety information.

Eyelash eyebrow serum	PG analogue name	Concentration of PG analogue disclosed? (Yes/No)	Eye side effects as reported from manufacturer
Latisse	Bimatoprost	Yes, 0.03%	“The most frequently reported adverse reactions were eye pruritus, conjunctival hyperemia, skin hyperpigmentation, ocular irritation, dry eye symptoms, and periorbital erythema. These reactions occurred in less than 4% of patients” [45]
Grande Lash MD	Isopropyl Cloprostenate	No, described as a “small amount”	“If redness, irritation or other unwanted effects occur, stop using product immediately” [30]
Rodan + Fields Lash Boost Eyelash Enhancing + Conditioning Serum	Isopropyl Cloprostenate	No	“Some users have reported eye redness and itchiness, dry eyes, watering of the eyes, sensitivity, styes, inflammation of eyelid and eye, temporary darkening of skin around the eye area, lash loss and/or visible enhancement of hair around or outside the eye area. Stop use immediately if you experience these or other symptoms” [46]
Revitalash	Cloprostenol Ethyl Amide	No	“A very small percentage of users reported sensitivity including temporary redness, swelling, itching, bumps, and burning sensation on the eyebrows. Even fewer users reported darkening of the skin at the eyebrows. It is not known whether RevitaBrow Advanced or some other factor caused the reported reactions, or some other factor. If this is a concern, do not use.” [47]
RapidLash	Isopropyl Cloprostenate	No	“Some users have reported temporary reactions, such as: eye redness and itchiness, dry eyes, watering of the eyes, blurry vision, sensitivity, styes, inflammation of eyelid and eye, darkening of skin around the eye area, lash loss and/or visible enhancement of hair around the or outside the eye area” [48]
Babe Lash	Isopropyl Cloprostenate	No	“If redness, irritation or other unwanted effects occur, stop using product immediately” [49]
NeuLash	Isopropyl Cloprostenate	No	“Some users have reported temporary reactions, such as: eye redness and itchiness, dry eyes, watering of the eyes, blurry vision, sensitivity, styes, inflammation of eyelid and eye, darkening of skin around the eye area, lash loss and/or visible enhancement of hair around the or outside the eye area. Stop use immediately if you experience these or other reactions” [50]

Certain patient factors have been linked to increased rates of PAP. Factors including older age and use of bimatoprost or travoprost have been found to be associated with the development of PAP, while higher body mass index was found to be protective against the development of PAP. Interestingly, the concurrent use of timolol was found to possibly precipitate periorbital change when used with PG analogues [31].

## 6 Diagnostic Evaluation and Differential Diagnosis

Recognition of PAP in dermatologic settings requires a high index of suspicion due to its ability to mimic more common periorbital changes. Clinically, PAP presents with periorbital fat loss, DUES, enophthalmos, eyelid retraction, and periorbital hollowing, features that easily mimic normal aging, previous cosmetic procedures, or other fat redistribution syndromes [21, 51, 52]. Additionally, the changes induced by prostaglandin analogues are often gradual and subtle, and patients rarely notice or report them, further increasing the likelihood of misattribution to aging or prior surgical interventions [21, 40]. In unilateral cases, the asymmetry may be attributed to previous cosmetic procedures or natural variation, unless a detailed medication history is obtained [40].

Several clinical features can assist in recognizing PAP. Unilateral application of prostaglandin analogues often produces asymmetrical periorbital changes, which may serve as a diagnostic clue [5]. In addition, distinct features of PAP, such as upper eyelid retraction rather than ptosis, may help distinguish it from the age‐related “sunken eyelid” appearance [21]. However, periocular changes associated with PAP may present variably across patients, and findings such as eyelid retraction or ptosis may coexist or differ in prominence, underscoring the importance of careful eyelid examination.

Distinguishing PAP from other causes of periorbital fat loss is critical for accurate diagnosis and appropriate management. HIV‐associated lipoatrophy can cause facial volume loss, but this is generally systemic. It manifests as generalized loss of subcutaneous adipose tissue, particularly in the extremities and buttocks, and is often accompanied by metabolic disturbances [53]. Thyroid eye disease (TED), especially in the late or quiescent phase, may present with similar features in terms of periorbital fat loss and eyelid contour changes. However, the changes observed in TED are often accompanied by a history of prior proptosis, extraocular muscle restriction, or other sequelae of orbital inflammation, and the changes are typically bilateral. PAP is usually unilateral or asymmetric, limited to the treated eye, and not associated with extraocular muscle involvement or a history of orbital inflammation [54]. PG analogues have been explored therapeutically in TED due to their potential to reduce soft tissue volume, potentially complicating the diagnostic picture [55]. Furthermore, surgical removal or repositioning of orbital fat can result in persistent or progressive periorbital hollowing, lid retraction, or asymmetry [56]. In such cases, a detailed history of periocular procedures is a key clue Box 1.

The following practical framework summarizes a stepwise clinical approach to recognizing, evaluating, and managing suspected PAP in dermatologic practice.

BOX 1 **Practical Dermatology‐Oriented Approach to Suspected Prostaglandin‐Associated Periorbitopathy.**

Recognize

- Periocular hollowing.
- DUES.
- Eyelid asymmetry or contour changes.
- Ptosis, enophthalmos.
- Periorbital hyperpigmentation or skin texture changes.

Ask

- Specific eyelash/eyebrow serum brand.
- Duration and frequency of use.
- Application site (lash line vs. eyebrows).
- Unilateral versus bilateral use.
- Concurrent ophthalmic prostaglandin therapy.

Act

- Discontinue or switch cosmetic prostaglandin‐containing products when appropriate.
- If glaucoma therapy is implicated, recommend coordination with ophthalmology rather than abrupt discontinuation.
- Counsel patients regarding the variable timeline and degree of potential reversibility.

## 7 Reversibility and Management

Once PAP is recognized, timely management is essential in preventing long‐term changes. Multiple studies suggest that early recognition can lead to partial or even near‐complete reversal of periorbital changes. One study revealed a reversal of periorbital hollowing just 6 months after discontinuing Lash Boost, with the patient reporting a 90%–95% return to her pre‐PG analogue appearance [5]. Another study followed nine patients with PAP and found that after 1 year of discontinuing PG analogue therapy there were improvements in eyelid sulcus depth, dermatochalasis, and infraorbital fat pads [57]. A representative image of PAP with subsequent improvement following discontinuation of therapy is shown in Figure 1. Additional studies have identified an impact of the specific PG analogue on the development or severity of PAP, with improvement in symptoms, reversal, or recovery observed after switching from bimatoprost to latanoprost [58, 59]. These studies suggest that there is a degree of reversibility for PAP. However, many factors contribute to the extent of recovery, including the duration of exposure and potency of the prostaglandin analogue used. To date, no studies have evaluated whether spacing out PG analogue application reduces the risk or severity of PAP, nor are there formal published recommendations for counseling patients to discontinue PG analogue use once adequate eyelash growth is achieved. These are important areas for future research aimed at improving PAP prevention and patient education.

**FIGURE 1:**
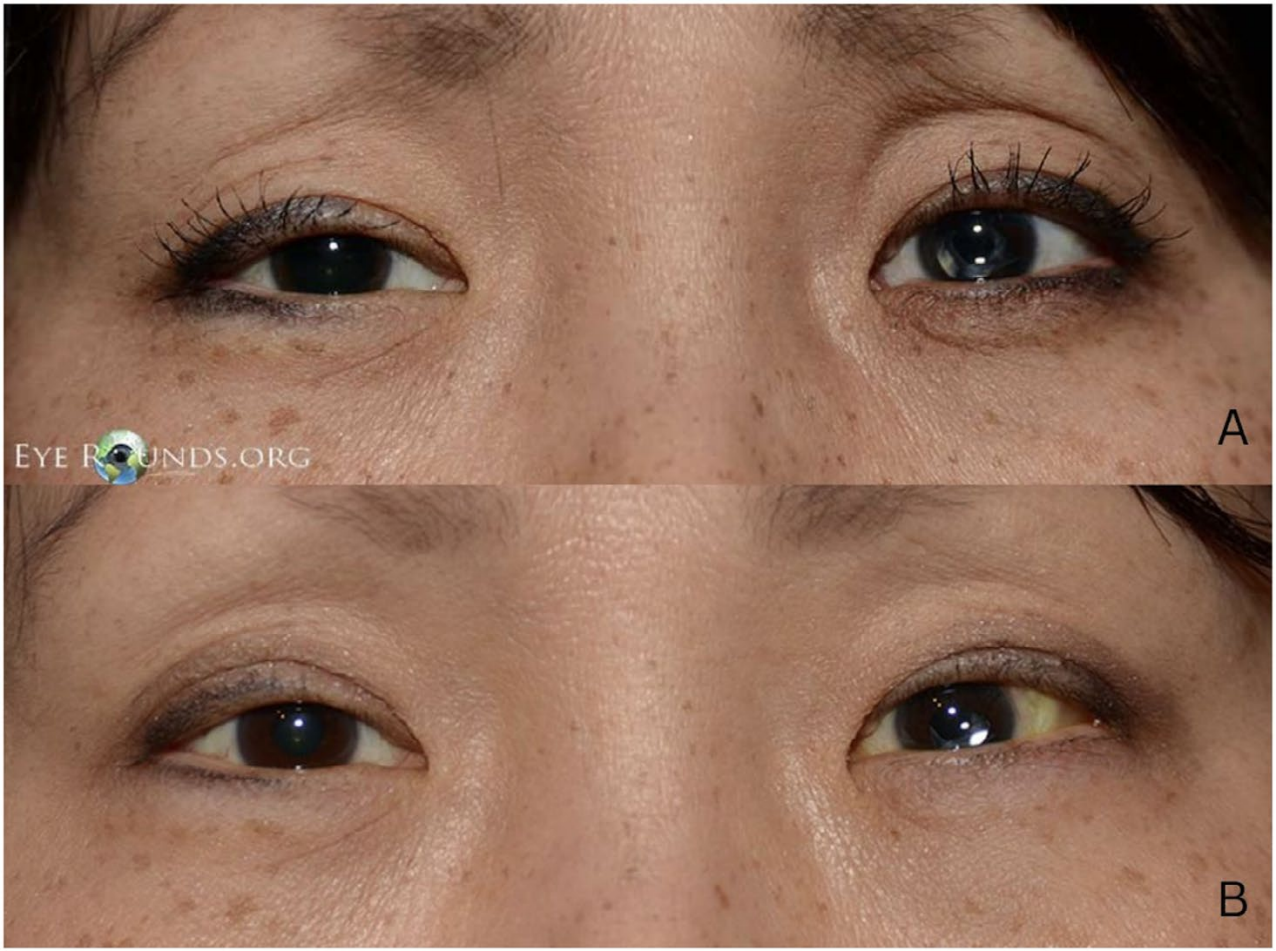
Prostaglandin‐Associated Periorbitopathy (PAP) with improvement after discontinuation. (A) A 52 years‐old female with angle recession glaucoma of the left eye treated with a prostaglandin analogue for many years demonstrates unilateral orbital fat atrophy, most notable in the superior sulcus. (B) One year after discontinuation of the prostaglandin analogue, there is marked improvement in orbital fat volume. Image source: Eyerounds.org, The University of Iowa. Available at: https://www.eyerounds.org/atlas/pages/PGA‐fat‐atrophy.htm.

Available evidence suggests that PAP may be at least partially reversible following discontinuation of PG analogue exposure; however, the degree and timeline of recovery appear highly variable. Factors such as duration of use, potency of the specific PG analogue, and individual patient characteristics likely influence the extent of improvement. Importantly, the current evidence base is limited and consists largely of case reports and small cohort studies, which restrict definitive conclusions regarding prognosis. Additionally, there are limited data evaluating dose spacing, maintenance regimens, or intermittent use, particularly in cosmetic settings, leaving uncertainty around strategies to mitigate risk while maintaining therapeutic or cosmetic benefit [5, 57, 58, 59].

For existing PAP with aesthetic or functional deformities, cosmetic correction may be another option. Some methods include autologous fat grafting to restore volume [60], dermal fillers to correct sulcus deepening [61], or even surgical intervention [62]. While published data on these methods for restoring periorbital fullness in PAP are limited, they may be particularly relevant in dermatologic practice with a focus on aesthetics and quality of life. Patient counseling regarding the risk of PAP for patients interested in using PG analogue‐containing eyelash serums is critical to prevent negative psychosocial consequences, particularly among patients with facial lipoatrophy, prior or intended eyelid surgery, or aesthetic goals [5, 27].

## 8 Prostaglandin Analogues in Eyelash Serums and Regulatory Considerations

Eyelash serums vary widely in their active ingredients, mechanisms of action, efficacy, and safety profiles. While topical bimatoprost is FDA‐approved for treating eyelash hypotrichosis, available evidence suggests that many OTC products contain PG analogues for which there are limited safety or efficacy data for cosmetic use. Under current U.S. regulations, cosmetics and OTC products are generally not subject to FDA review, placing the burden of risk assessment on consumers and clinicians. Mass spectrometry analyses have reported the presence of undisclosed PG analogues, including bimatoprost and latanoprost, in several OTC eyelash serums, despite their absence from product labeling. This regulatory landscape may increase the risk of unintentional exposure to pharmacologically active agents with potential side effects [63, 64]. Incomplete or variable labeling may limit patient awareness and increase the likelihood of adverse effects [4].

The FDA has issued warnings regarding bimatoprost side effects, including pruritus, irritation, and hyperpigmentation. Bimatoprost is not recommended for use during pregnancy or while breastfeeding due to its detection in breast milk, and latanoprost has been found to have a similar side effect profile [64]. These considerations highlight the importance of accurate labeling and regulatory oversight to support safety monitoring and informed use.

Due to the lack of ingredient transparency among OTC eyelash and eyebrow enhancement serums, dermatologists should thoroughly document both medications and cosmetic products used by their patients, specifically inquiring about eyelash and eyebrow serums. Dermatologists should also counsel patients regarding potential side effects and long‐term implications of serums that may contain PG analogues.

## 9 Gaps in Literature and Research Needs

The existing literature on PG analogue‐associated cutaneous and hair effects is heterogeneous and largely consists of case reports, small cohort studies, and ophthalmology‐focused trials, which limits formal assessment of evidence quality. Underreporting of adverse effects of PG analogues is a well‐recognized issue, particularly regarding industry‐sponsored trials. A literature review focusing on bimatoprost as a treatment for eyelash hypotrichosis found that the majority of literature comes from industry‐sponsored clinical trials. This particular study design can facilitate systemic underreporting of adverse effects of bimatoprost, including discomfort with application and PAP, and a lack of knowledge regarding increased iris pigmentation [5]. Further investigation and education are needed regarding the adverse effects of PG analogues.

There is also a gap in dermatology‐specific knowledge regarding PAP. The literature surrounding adverse effects of PG analogues is largely driven by ophthalmology‐focused studies, primarily in the context of PG analogue use as a treatment for glaucoma or ocular hypotension [1, 6, 7, 9, 10, 11, 12, 13, 14, 15, 16, 17, 18, 19, 21, 22, 24, 25, 26]. In these settings, the benefits of PG analogue use for preserving vision may outweigh the risk of local side effects like PAP. However, in dermatologic or cosmetic contexts, where treatment goals are aesthetic or adjunctive, the risk–benefit balance may shift, making potential adverse effects less acceptable. Compounding this concern, existing literature on PAP often consists of case reports or small cohort studies rather than systematic evaluations or randomized controlled trials [6, 9, 10, 11, 12, 13, 16, 17, 18, 19, 20, 21, 22, 25, 26]. In combination with underreporting of adverse events, the lack of dermatology‐focused research underscores the need for continued investigation and careful patient counseling.

There are currently no standardized clinical guidelines governing the use of PG analogues in dermatology. Specifically, there is an absence of guidelines for off‐label uses of PG analogues in hair disorders such as alopecia. A systematic review of topical PG analogue usage in hair loss noted the efficacy of the analogues but found that the best dosage and frequency require further studies to be determined [65]. With its increasing usage in dermatology, it is critical to establish standardized and uniform guidelines to ensure the safest and most effective usage for patients.

## 10 Conclusion

As PG analogues gain popularity in dermatology for both cosmetic enhancement and alopecia management, their potential to cause periorbital fat atrophy and associated structural changes requires greater clinical awareness. Although these agents offer clear benefits in stimulating eyelash and eyebrow growth, even short‐term or over‐the‐counter use can lead to distressing aesthetic effects. Given the growing evidence of underrecognized adverse outcomes, compounded by regulatory gaps and underreporting, it is essential that dermatologists take a proactive role in patient education, obtain thorough histories including cosmetic product use, and advocate for improved labeling standards. Future research focused specifically on dermatologic applications is critical to better define the safety profile, dosing parameters, and long‐term effects of PG analogues, and to inform the development of evidence‐based guidelines for their use.

## Author Contributions

All authors contributed substantially to the conception and design of the work, critically revised the manuscript for important intellectual content, approved the final version, and agree to be accountable for all aspects of the work.

## Disclosure

AI use statement: AI‐assisted tools were not used in the preparation, writing, or editing of this manuscript.

## Ethics Statement

The authors confirm that the ethical policies of the journal, as noted on the journal's author guidelines page, have been adhered to. No ethical approval was required as this is a review article with no original research data.

## Consent

The image is publicly available for educational use through the source institution, and formal permission for reuse/publication was obtained from the copyright holder.

## Conflicts of Interest

The authors declare no conflicts of interest.

## Data Availability

Data sharing not applicable to this article as no datasets were generated or analysed during the current study.
